# Prediction of properties of some drugs used in the treatment of bipolar disorder via various Zagreb indices

**DOI:** 10.1038/s41598-026-58293-5

**Published:** 2026-06-18

**Authors:** Özge Çolakoğlu, Alaa Altassan

**Affiliations:** 1 Mathematics Department, Science Faculty, Mersin University, 33343 Mersin, Turkey; 2 Department of Mathematics, Faculty of Science, King Abdulaziz University, Jeddah, 24123 Saudi Arabia

**Keywords:** Topological indices, Zagreb indices, Bipolar disorder drugs, QSPR, Chemistry, Mathematics and computing

## Abstract

Predicting physicochemical properties of chemical compounds in a cost-effective and non-experimental manner is of great importance. This is often achieved using topological indices derived from graph theory. Topological index is the numerical value obtained from the structural property of the graph obtained by modeling the chemical structure with graph theory. Bipolar disorder is a mental health condition characterized by mood swings, including manic/hypomanic and depressive episodes. In this study, various Zagreb topological indices based on vertex degree, edge degree, and eccentricity are calculated for graphs of drugs used in the treatment of bipolar disorder. Furthermore, QSPR (quantitative structure property relationship) models are developed to predict the boiling point, enthalpy of vaporization, flash point, molar refractivity and polarizability of these drugs. This study determines the version of the Zagreb indices that best predict the physicochemical properties of bipolar drugs and the corresponding model. The findings demonstrate the potential of using mathematical descriptors in designing and evaluating pharmaceutical compounds.

## Introduction

Chemical graph theory focuses on the mathematical modeling of a chemical compound^1^. The molecular graph of a chemical compound is constructed by representing atoms as vertices and bonds as edges. Topological indices are numerical descriptors based on the structure of the molecular graph and are typically categorized as degree-based, edge-based, or distance-based^2^. Topological indices (graph indices) are used to estimate the physical/chemical properties and biological activities of the chemical in QSPR/QSAR studies. The first application of graph index was studied by H. Wiener in 1947. It has been used to determine some physical properties of paraffins^3^. There are many graph indices in the literature and new ones are added every day. There are no definite criteria yet to stop or slow down these new indices. However, by comparing these indices, it is possible to obtain indices that are more valuable and have better predictive ability. Among topological indices, Zagreb indices are some of the most widely studied due to their simplicity and applicability (see^4–6^). Over time, various extensions and modifications of Zagreb indices have been proposed, including reformulated versions, eccentricity-based versions, and two-distance degree-dependent variants.

Hakeem et al. used QSPR models with topological indices for predicting of physicochemical properties of bioactive polyphenols^7^. Mahboob et al. studied Zagreb indices and linear regression models for estimate properties of anti-hepatitis drugs^8^. Jamal et al. examined regression models with degree-based indices of some properties of alkaloids^9^. Saleh et al. obtained QSPR models and calculated topological indices of some chemical structures^10^. Ali et al. studied on various Zagreb indices and chemical trees^11^. Horoldagva et al. given results on Zagreb indices of graphs^12^.

In drug discovery, designing drugs in computational environments and predicting their properties without conducting experiments is becoming increasingly important. Numerous studies exist on this topic^13–15^. Graph models of drugs used in the treatment of many diseases have been obtained, their topological indices calculated, and regression models for this group of drugs have been derived. Alam et al. calculated the topological indices of drugs used against tuberculosis and obtained QSPR models^16^. There are many types of cancer. Many scientists have modeled the drugs used against these diseases using graphs and obtained QSPR models for the physicochemical properties of these drugs (see^17–21^). QSPR regression models have been obtained using various topological indices for drugs used against COVID-19, a disease as serious as cancer(^22–24^).However, there are currently no studies in the literature on medications used in the treatment of bipolar disorder.

Bipolar disorder is a major health problem worldwide. Bipolar disorder typically involves mood swings, ranging from mania or hypomania to severe depression, mixed states, or rapid cycling, The mechanisms of action of effective anti-bipolar disorder medications are not yet fully understood^25^. It can cause significant impairments in various aspects of life and affects approximately 40 million people worldwide. Medications for bipolar disorder are used to treat the symptoms of mania, hypomania, and depression experienced by people with bipolar disorder. Bipolar disorder cannot be cured, but several medications are approved by FDA to help people manage their symptoms^26^.

**Fig. 1:**
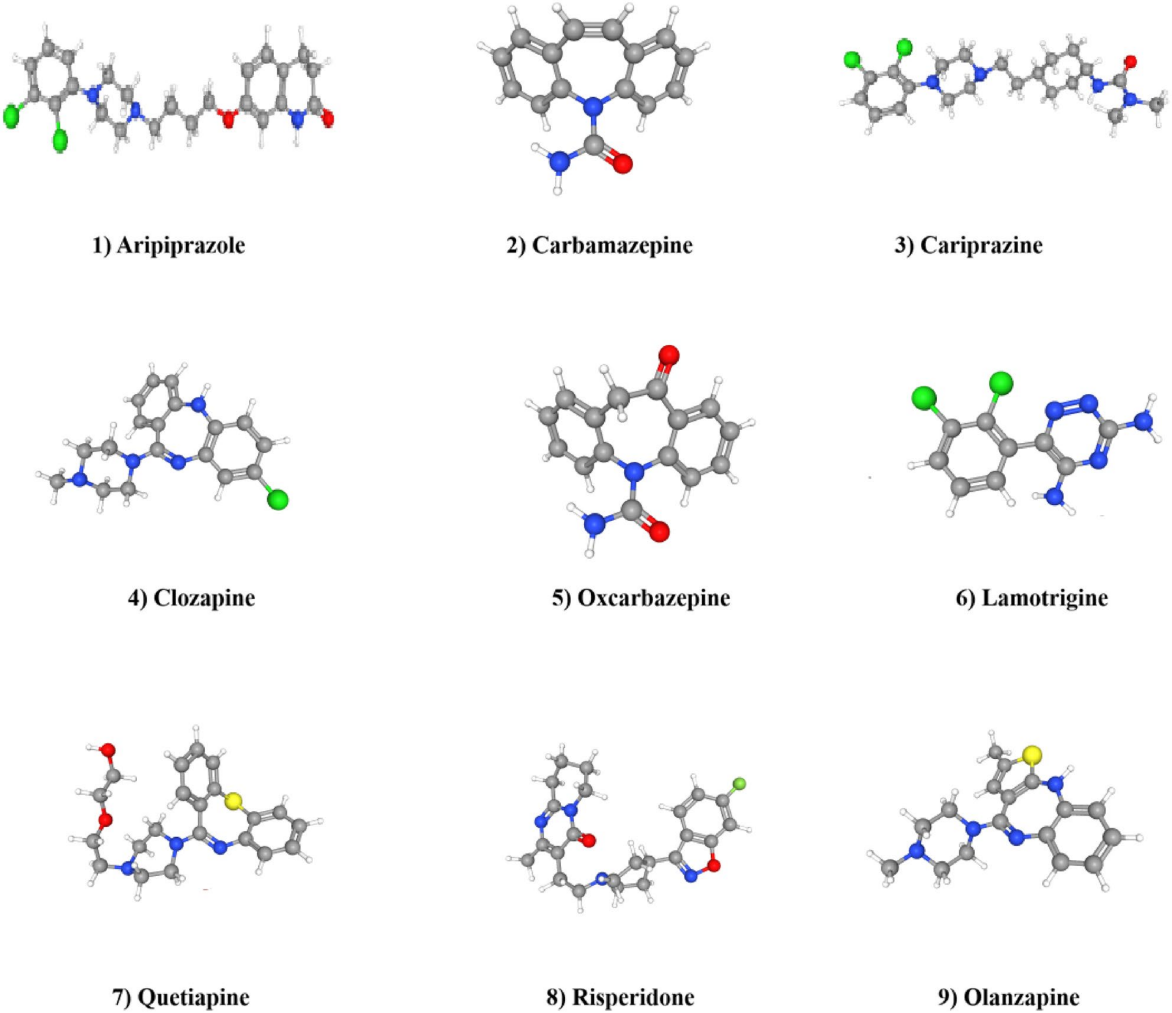
Chemical structure of drugs used in the treatment of bipolar disorder.

This study focuses on the drugs Aripiprazole, Carbamazepine, Cariprazine, Clozapine, Oxcarbazepine, Lamotrigine, Quetiapine, Risperidone and Olanzapine, which are a representative subset of commonly prescribed and structurally diverse drugs and used in the treatment of bipolar disorder. Comparison sets and figures of chemical structural properties of drugs used in the treatment of bipolar disorder were taken from PubChem and ChemSpider. The chemical structures of these drugs are given in Figure 1.

Table 1 shows the boiling point (BP), enthalpy of vaporization (EV), Flash Point (FP), Molar Refractivity (MR) and Polarizability (P) of these drugs.

**Table 1:** The properties of drugs used in the treatment of bipolar disorder.

Drugs No	Drugs Name	BP	EV	FP	MR	P
1	Aripiprazole	646,2	95,3	344,6	120,3	47,7
2	Carbamazepine	411	66,3	202,4	60,7	27,6
3	Cariprazine	600,1	89,3	316,7	117,2	46,4
4	Clozapine	489,2	75,5	249,6	93,7	37,2
5	Oxcarbazepine	457,2	71,7	230,3	70,2	27,8
6	Lamotrigine	503,1	77,2	258,1	63,4	25,1
7	Quetiapine	556,5	88,2	290,4	110,2	43,7
8	Risperidone	572,4	85,8	300	111,7	44,3
9	Olanzapine	476	74	241,7	92,2	36,5

In the present work, these drugs are modeled with graph theory, and their topological indices are calculated for use in QSPR analysis. These indices are various versions of the first and second Zagreb indices, which have significant importance in the literature. Specifically, it aims to predict the boiling points, enthalpy of vaporization, flash point, molar refractivity and polarizability of drugs used in the treatment of bipolar disorder by applying linear, logarithmic, quadratic, and cubic regression models using these indices. The best-performing models and indices will be identified and discussed.

## Materials and methods

Each chemical compound is represented as a simple undirected graph ($\Gamma (V (\Gamma ),E(\Gamma ))$), where vertices correspond to atoms and edges correspond to chemical bonds between atoms. Hydrogen atoms were omitted for simplification, and only heavy atoms are considered in constructing the molecular graphs^27^.The degree of a vertex $u \in V(\Gamma )$, denoted *d*(*u*), is the number of edges incident to *u*. The 2-distance degree of a vertex *v* in $\Gamma$ is defined by $d_2(v)$ and is defined as the number of vertices which are at distance two from the vertex *v* in $\Gamma$. The distance between vertices of *s* and *t* is denoted by *d*(*s*, *t*). The degree of an edge, *d*(*e*) may be defined in terms of the degrees of its adjacent vertices i.e. $d(e)=d(u)+d(v)-2$ for $e=uv\in E(\Gamma )$. The eccentricity $\epsilon (v)$ of a vertex is the greatest distance between *v* and any other vertex in the graph^28^. If edges *e* and *f* are adjacent, it is denoted by $e \sim f$.

The first $(M_{1} ({\Gamma }))$ and second Zagreb ($M_{2} ({\Gamma })$) indices were introduced by Gutman and Trinajstic in 1972^29^. In 2003, Nikolic et al. introduced modified versions of these indices^30^. Milicevic et al. (2004) developed the reformulated Zagreb indices, which focus on edge contributions^31^. The leap expansion of these indices was defined in 2017^32^, the eccentric expansion in 2012^33^. In 1997, Sharma, Goswami, and Madan proposed the eccentric connectivity index^34^, and its edge-based version was later defined by Xu and Guo in 2012^35^. Moreover, the leap extension of eccentric connectivity index has been defined by many authors^36,37^.

With the motivation of the above authors, the edge version of the eccentric Zagreb indices are defined as follows:

$$EM_1^* (\Gamma )$$= $$\sum _{e \sim f } {\epsilon (e) + \epsilon (f)}$$

and

$$EM_2^* (\Gamma )$$= $$\sum _{e \sim f } {\epsilon (e) \epsilon (f)}$$.

Table 2 shows mathematical expressions of graph indices.

**Table 2:** Various topological indices derived from $M_1(\Gamma )$ and $M_2(\Gamma )$.

Topological Indices	Expressions
First Zagreb Index^29^	$M_1(\Gamma )$=$\sum _{\tau q \in E(\Gamma )} (d(\tau ) +d(q))$
Second Zagreb Index^29^	$M_2(\Gamma )$=$\sum _{\tau q \in E(\Gamma )} (d(\tau ) d(q))$
Redefined First Zagreb Index^38^	$ReM_1(\Gamma )$=$\sum _{\tau q \in E(\Gamma )} \frac{d(\tau )+ d(q)}{d(\tau ) d(q)}$
Redefined second Zagreb Index^38^	$ReM_2(\Gamma )$=$\sum _{\tau q \in E(\Gamma )} \frac{d(\tau ) d_q}{d(\tau ) +d(q)}$
Modified First Zagreb Index^30^	$MoM_1 (\Gamma )$= $\sum _{\tau q \in E(\Gamma )} \frac{1}{ d(q)}$
Modified Second Zagreb Index^30^	$MoM_2 (\Gamma )$= $\sum _{\tau q \in E(\Gamma )} \frac{1}{d(\tau ) d(q)}$
Reformulated first Zagreb Index^31^	$RM_1 (\Gamma )$= $\sum _{e \in E(\Gamma )} {(d(e))^2}$
Reformulated Second Zagreb Index^31^	$RM_2 (\Gamma )$= $\sum _{e \sim f } {(d(e) d(f))}$
First leap Zagreb Index^32^	$LM_1 (\Gamma )$= $\sum _{\tau q \in E(\Gamma )} {d_2 (\tau ) + d_2 (q)}$
Second leap Zagreb Index^32^	$LM_2 (\Gamma )$= $\sum _{\tau q \in E(\Gamma )} {d_2 (\tau ) d_2 (q)}$
Eccentric first Zagreb index^33^	$M_1^* (\Gamma )$= $\sum _{\tau q \in E(\Gamma )} {\epsilon (\tau ) + \epsilon (q)}$
Eccentric second Zagreb index^33^	$M_2^* (\Gamma )$= $\sum _{\tau q \in E(\Gamma )} {\epsilon (\tau ) \epsilon (q)}$
Eccentric first Zagreb index (Different Version)^33^	$$M_1^{**} (\Gamma )$$= $$\sum _{\tau \in V(\Gamma )} {\epsilon (\tau ) ^2}$$
Edge eccentric first Zagreb index	$EM_1^* (\Gamma )$= $\sum _{e \sim f } {(\epsilon (e) + \epsilon (f))}$
Edge eccentric second Zagreb index	$EM_2^* (\Gamma )$= $\sum _{e \sim f } {(\epsilon (e) \epsilon (f))}$
Eccentric Connectivity Index^34^	$\xi ^ c(\Gamma )$= $\sum _{\tau \in V({\Gamma })} d_\tau \epsilon ( \tau )$
Edge Eccentric Connectivity Index^35^	$\xi _e ^ c(\Gamma )$= $\sum _{e \in E(\Gamma )} d_e \epsilon (e)$
Leap Eccentric Connectivity Index^36,37^	$L\xi ^ c(\Gamma )$= $\sum _{\tau \in V({\Gamma })} d_2(\tau ) \epsilon ( \tau )$

## Regression models

This section discusses the method to be used in the analysis of graph indices obtained for bipolar drugs. Here, curvilinear and nonlinear regression models will be generated using SPSS (IBM Statistics 20 license). The use of multiple regression models (linear, logarithmic, second-order, and third-order) allows us to capture both linear and nonlinear relationships between topological indices and physicochemical properties; therefore, multiple models were considered to determine the best prediction performance. These models are:

$$\begin{aligned} y&=a+b\tilde{\xi } \\ y&=a+b \ln {\tilde{\xi }} \\ y&=a+b\tilde{\xi }+c\tilde{\xi }^{2} \\ y&=a+b\tilde{\xi }+c\tilde{\xi }^{2}+d\tilde{\xi }^{3}, \end{aligned}$$

where *y* is the property of chemical structure, *a* is constant, *b*, *c*, *d* are the coefficients for the graph index, and $\tilde{\xi }$ are graph indices. *R* is the correlation coefficient. These models were selected to capture both linear and nonlinear relationships between descriptors and properties. QSPR relationships are often nonlinear; therefore, using multiple regression forms provides a more comprehensive analysis.

If the theoretical result and the experimental result are close to each other, the correlation coefficient is close to 1. The closeness of the theoretical and experimental results to each other increases the prediction quality of the model. In this study, for the predictive ability and quality of the model will be discussed measures the maximum correlation and minimum root mean square error (RMSE). The root mean square error is defined as

$$\begin{aligned} RMSE=\sqrt{\dfrac{\sum _{k=1}^{n}(\rho _{k}-\vartheta _{k})^{2}}{n}}, \end{aligned}$$

where $\rho _{k}$ is the observed value of chemical properties, $\vartheta _{k}$ is predicted value, *n* is the samples number in the test^39^.

## Main results

The values of the topological indices were computed using a combination of manual derivations and computational tools. The structural parameters of each molecular graph were first identified, and then the corresponding indices were calculated using their mathematical definitions. The results in Table 3 are obtained from Table 2 and molecular graphs of chemical compounds in Figure 1. Table 3 shows the values of topological indices of drugs used in the treatment of bipolar disorder.

**Table 3:** Topological indices of drugs used in the treatment of bipolar disorder.

no	1	2	3	4	5	6	7	8	9
$M_{1}$	156	96	136	114	102	82	140	166	122
$M_{2}$	181	115	154	139	123	96	164	200	146
$ReM_{1}$	30	18	27.333	20	19	16	27	30	22
$ReM_{2}$	37.45	23.2	32.05	27.4	24.45	19.2	34.266	39.95	29.3
$MoM_{1}$	7.1166	4.2833	6.3	7.733	4.45	3.616	6.6	7.1166	5.2166
$MoM_{2}$	6.6111	3.9722	6.0833	4.733	4.166	3.527	6.11	6.444	4.694
$RM_{1}$	259	170	238	212	186	146	230	297	218
$RM_{2}$	375	266	343	336	296	222	341	454	334
$LM_{1}$	427	320	401	403	346	248	440	486	410
$LM_{2}$	1504	1350	1384	1881	1507	937	1814	2250	1725
$M_{1}^*$	949	232	793	292	234	207	691	887	377
$M_{2}^*$	7018	619	5407	904	661	644	4099	5946	1450
$$M_{1}^**$$	6490	603	5263	803	639	659	3805	5367	1350
$EM_{1}^*$	1257	283	1039	391	290	658	865	1216	479
$EM_{2}^*$	9007	698	6794	1146	791	728	4823	7656	1673
$\xi ^ c$	949	232	793	280	234	207	717	887	377
$\xi _e ^ c$	1257	294	1039	391	314	261	865	1216	497
$L\xi ^ c$	2890	736	2407	982	792	665	2128	3021	1246

Tables 1 and 3 show the correlations between the topological index and the properties of the drugs in linear, quadratic, third-order, and logarithmic regression models. The closest correlation to one for each property is marked in bold. Table 4 shows the correlation coefficients of the regression models for boiling point.

**Table 4:** Correlations between TI and the BP feature.

** TI**	Linear	Logarithmic	Quadratic	Cubic
$M_{1}$	0.788	0.763	0.800	0.800
$M_{2}$	0.732	0.709	0.736	0.740
$ReM_{1}$	0.721	0.705	0.722	0.722
$ReM_{2}$	0.611	0.600	0.611	0.613
$MoM_{1}$	0.628	0.630	0.647	0.661
$MoM_{2}$	0.869	0.842	0.921	0.919
$RM_{1}$	0.859	0.836	0.892	0.888
$RM_{2}$	0.768	0.739	0.785	0.785
$LM_{1}$	0.560	0.524	0.614	0.603
$LM_{2}$	0.189	0.179	0.200	0.250
$M_{1}^*$	0.921	0.902	0.923	0.923
$M_{2}^*$	0.931	0.912	0.932	0.935
$$M_{1}^**$$	0.936	0.917	0.936	0.941
$EM_{1}^*$	**0.950**	**0.945**	**0.952**	**0.952**
$EM_{2}^*$	0.930	0.916	0.930	0.935
$\xi ^ c$	0.915	0.893	0.920	0.920
$\xi _e ^ c$	0.909	0.888	0.909	0.913
$L\xi ^ c$	0.891	0.879	0.892	0.916

The indices and models with the best predictive ability for the BP feature in linear, logarithmic, quadratic, and cubic models, based on the correlations given in Table 4, are presented in Table 5.

**Table 5:** Model performance metrics for different regression models for BP feature.

Models	Equations	$R^2$	RMSE
Linear	$BP = 392.673 + 0.183EM_1^*$	0.903	25.069
Logarithmic	$BP = -225.970 + 116.717 \ln (EM_1^*)$	0.894	26.214
Quadratic	$BP = 375.396 + 0.243EM_1^* - 3.991\times 10^{-5}(EM_1^*)^2$	0.906	26.700
Cubic	$BP = 343.487 + 0.414EM_1^* + 0.000(EM_1^*)^2 + 1.132\times 10^{-7}(EM_1^*)^3$	0.907	29.028

Table 6 shows the correlation coefficients of the regression models for Enthalpy of Vaporization.

**Table 6:** Correlations between TI and the EV feature.

TI	Linear	Logarithmic	Quadratic	Cubic
$M_{1}$	0.793	0.773	0.799	0.801
$M_{2}$	0.737	0.719	0.738	0.738
$ReM_{1}$	0.870	0.850	0.891	0.887
$ReM_{2}$	0.780	0.754	0.790	0.790
$MoM_{1}$	0.634	0.639	0.663	0.680
$MoM_{2}$	0.886	0.861	0.929	0.926
$RM_{1}$	0.711	0.700	0.711	0.713
$RM_{2}$	0.598	0.592	0.601	0.606
$LM_{1}$	0.581	0.546	0.630	0.619
$LM_{2}$	0.211	0.205	0.213	0.232
$M_{1}^*$	0.922	0.913	0.923	0.923
$M_{2}^*$	0.926	0.923	0.928	0.934
$$M_{1}^**$$	0.930	0.927	0.931	0.938
$EM_{1}^*$	**0.938**	**0.941**	** 0.944**	** 0.945**
$EM_{2}^*$	0.919	0.923	0.925	0.935
$\xi ^ c$	0.921	0.906	0.921	0.922
$\xi _e ^ c$	0.904	0.894	0.906	0.914
$L\xi ^ c$	0.889	0.887	0.898	0.930

From Table 6, the indices and linear, logarithmic, quadratic, and cubic models with the best predictive ability for the EV feature are given below (see Table 7):

**Table 7:** Model performance metrics for different regression models for the EV feature.

Models	Equations	$R^2$	RMSE
Linear	$EV = 63.849 + 0.023EM_1^*$	0.880	3.792
Logarithmic	$EV = -15.033 + 14.856 \ln (EM_1^*)$	0.885	3.857
Quadratic	$EV = 59.154 + 0.039EM_1^* - 1.085E-005(EM_1^*)^2$	0.892	4.054
Cubic	$EV = 61.169 + 0.029EM_1^* + 5.348E-006(EM_1^*)^2 -7.149E-009(EM_1^*)^3$	0.892	4.207

Table 8 shows the correlation between the indices and the flash point feature.

**Table 8:** Correlations between TI and the FP property.

TI	Linear	Logarithmic	Quadratic	Cubic
$M_{1}$	0.788	0.763	0.800	0.800
$M_{2}$	0.732	0.709	0.736	0.740
$ReM_{1}$	0.859	0.836	0.893	0.889
$ReM_{2}$	0.768	0.739	0.785	0.785
$MoM_{1}$	0.628	0.630	0.647	0.661
$MoM_{2}$	0.869	0.842	0.922	0.919
$RM_{1}$	0.721	0.705	0.722	0.722
$RM_{2}$	0.611	0.600	0.611	0.613
$LM_{1}$	0.559	0.524	0.614	0.603
$LM_{2}$	0.189	0.179	0.200	0.250
$M_{1}^*$	0.921	0.903	0.923	0.923
$M_{2}^*$	0.931	0.913	0.932	0.935
$$M_{1}^**$$	0.936	0.917	0.937	0.941
$EM_{1}^*$	** 0.950**	**0.946**	**0.952**	**0.953**
$EM_{2}^*$	0.930	0.916	0.930	0.935
$\xi ^ c$	0.915	0.893	0.920	0.920
$\xi _e ^ c$	0.909	0.888	0.909	0.913
$L\xi ^ c$	0.891	0.879	0.892	0.916

Table 9 shows the models of indices in Table 8 that have the closest correlation to 1 for each regression model for the FP feature.

**Table 9:** Model performance metrics for different regression models for the FP property.

Models	Equations	$R^2$	RMSE
Linear	$FP = 191.287 + 0.110EM_1^*$	0.903	15.135
Logarithmic	$FP = -182.856 +70.586 \ln (EM_1^*)$	0.894	15.827
Quadratic	$FP = 180.811 + 0.147EM_1^* -(2.420E+005)(EM_1^*)^2$	0.906	16.118
Cubic	$FP = 161.561 + 0.250EM_1^* + 0.000(EM_1^*)^2 + 6.829E-008(EM_1^*)^3$	0.907	17.524

Table 10 shows the correlation between the indices and the molar refractivity property.

**Table 10:** Correlations between TI and the MR property.

TI	Linear	Logarithmic	Quadratic	Cubic
$M_{1}$	0.921	0.934	0.944	0.947
$M_{2}$	0.889	0.905	0.919	0.924
$ReM_{1}$	0.942	0.951	0.957	0.958
$ReM_{2}$	0.913	0.923	0.931	0.935
$MoM_{1}$	0.841	0.865	0.917	0.928
$MoM_{2}$	** 0.967**	** 0.968**	** 0.971**	0.972
$RM_{1}$	0.888	0.906	0.924	0.930
$RM_{2}$	0.816	0.839	0.862	0.869
$LM_{1}$	0.851	0.838	0.851	0.851
$LM_{2}$	0.537	0.557	0.561	0.568
$M_{1}^*$	0.924	0.961	0.963	**0.980**
$M_{2}^*$	0.894	0.948	0.928	0.958
$$M_{1}^**$$	0.894	0.939	0.922	0.947
$EM_{1}^*$	0.825	0.822	0.826	0.826
$EM_{2}^*$	0.887	0.954	0.931	0.965
$\xi ^ c$	0.919	0.951	0.952	0.969
$\xi _e ^ c$	0.914	0.956	0.964	0.975
$L\xi ^ c$	0.910	0.954	0.968	0.974

The model of the index with the closest correlation coefficient to 1 in Table 10 for the molar refractivity property, along with the square of the correlation coefficient and the RMSE values, are given in Table 11.

**Table 11:** Model performance metrics for different regression models for MR.

Models	Equations	$R^2$	RMSE
Linear	$MR = -6.076 + 19.298MoM_2$	0.926	6.855
Logarithmic	$MR = -64.138 +97.478 \ln (MoM_2)$	0.938	6.261
Quadratic	$MR = -102.784 + 58.677MoM_2 -(3.824)(MoM_2)^2$	0.944	6.443
Cubic	$MR = -27.579 + 0.601M_1^* - 0.001(M_1^*)^2 + 4.174E-007(M_1^*)^3$	0.961	5.844

Table 12 shows the correlation between the indices and the polarizability property.

**Table 12:** Correlations between TI and the P property.

TI	Linear	Logarithmic	Quadratic	Cubic
$M_{1}$	0.930	0.943	0.953	0.957
$M_{2}$	0.898	0.915	0.929	0.934
$ReM_{1}$	0.952	0.961	0.967	0.967
$ReM_{2}$	0.923	0.935	0.944	0.947
$MoM_{1}$	0.843	0.870	0.928	0.939
$MoM_{2}$	** 0.972**	** 0.979**	** 0.982**	** 0.982**
$RM_{1}$	0.893	0.912	0.930	0.935
$RM_{2}$	0.819	0.844	0.868	0.874
$LM_{1}$	0.858	0.849	0.858	0.858
$LM_{2}$	0.540	0.566	0.572	0.592
$M_{1}^*$	0.932	0.965	0.965	0.978
$M_{2}^*$	0.905	0.954	0.936	0.960
$$M_{1}^**$$	0.905	0.945	0.929	0.950
$EM_{1}^*$	0.818	0.801	0.818	0.819
$EM_{2}^*$	0.898	0.959	0.938	0.967
$\xi ^ c$	0.930	0.959	0.958	0.973
$\xi _e ^ c$	0.924	0.964	0.970	0.980
$L\xi ^ c$	0.919	0.961	0.974	0.977

Table 13 shows the best predictive indices, models, and their model evaluation parameters from the correlations in Table 12 for the P feature.

**Table 13:** Model performance metrics for different regression models.

Models	Equations	$R^2$	RMSE
Linear	$P = -0.108 + 7.278MoM_2$	0.946	2.183
Logarithmic	$P = -22.012 +30.767 \ln (MoM_2)$	0.958	1.908
Quadratic	$P = -35.801 + 21.812MoM_2 -(1.412)(MoM_2)^2$	0.963	1.936
Cubic	$P = -25.429 + 15.101MoM_2 -0.096(MoM_2)^3$	0.964	1.913

**Fig. 2:**
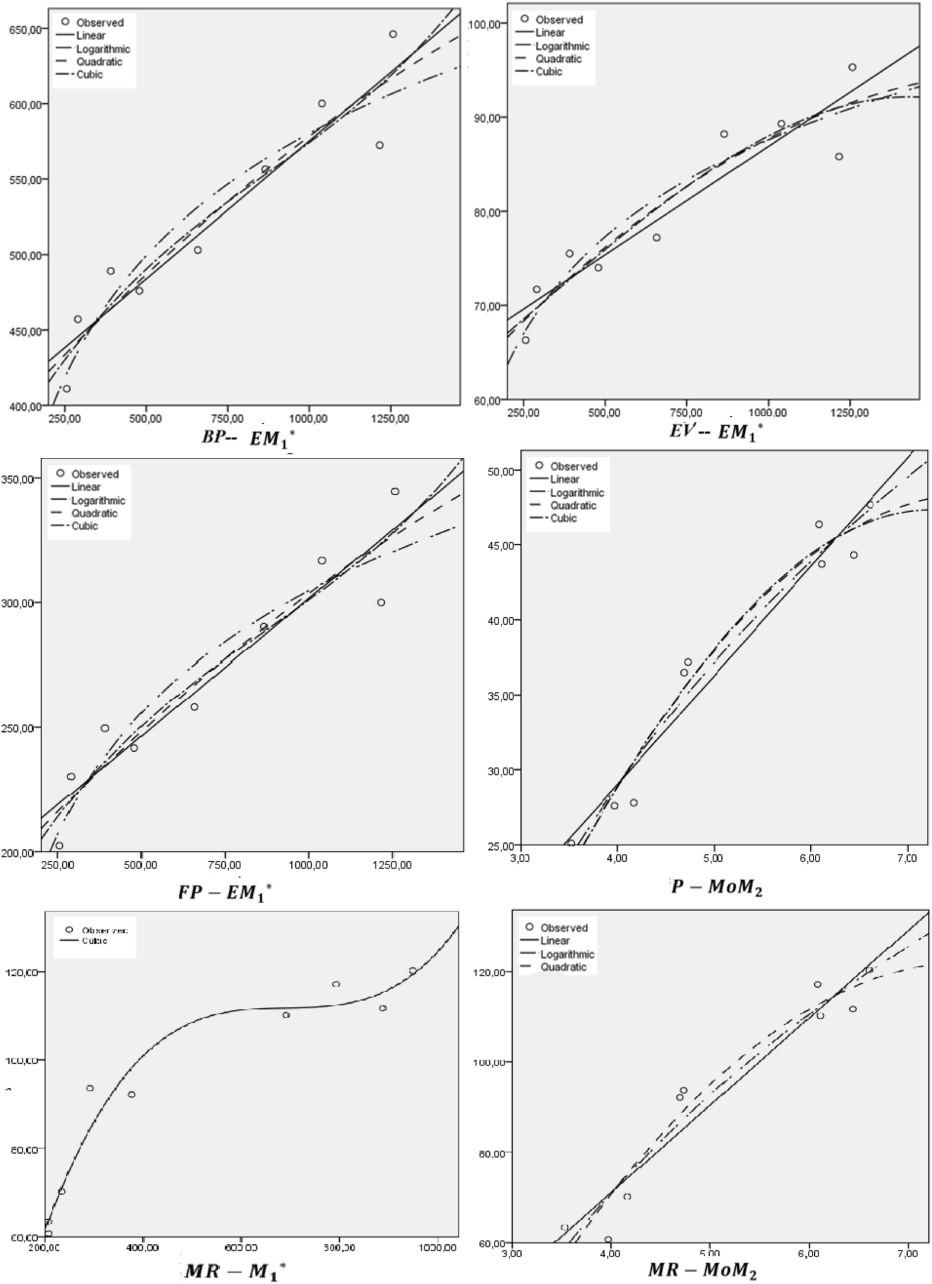
Comparing their actual values with the values of the models that best predict of drugs used in the treatment of bipolar disorder.

Figure 2 shows graphical illustrating the properties of medications used in the treatment of bipolar disorder, comparing their actual values with the values of the models that best predict them.

The results indicate that different topological indices exhibit varying predictive capabilities depending on the physicochemical property under consideration. Among all indices, the edge eccentric first Zagreb index ($EM_{1}^*$) consistently provides the highest correlation values for boiling point, enthalpy of vaporization, and flash point. This strong performance can be attributed to the fact that $EM_{1}^*$ incorporates both connectivity and distance-related structural information. Since physicochemical properties such as boiling point and enthalpy are influenced by molecular size, branching, and spatial distribution, indices that encode both local and global structural characteristics tend to perform better.

On the other hand, for molar refractivity (MR) and polarizability (P), the modified second Zagreb index ($MoM_{2}$) shows superior predictive performance. These properties are closely related to electron distribution and molecular volume, which are effectively captured by degree-based descriptors.

The comparison of regression models shows that cubic models generally yield the highest ($R^{2}$) values, indicating that the relationship between topological indices and physicochemical properties is nonlinear. However, the improvement over quadratic models is sometimes marginal, suggesting that simpler models may still be preferable in practical applications. Overall, the findings confirm that topological indices, particularly Zagreb-type indices, are effective tools for QSPR modeling and can provide meaningful insights into the structural determinants of physicochemical properties.

## Conclusions

With advancements in technology and science, efforts are being made to find solutions to diseases. Today, computer-aided drug discovery, which does not require experimentation, saves both time and money. One method is to model existing drugs using graphs, obtain numerical results from them, and derive equations that relate these results to experimental results. These equations can be used to predict the physicochemical properties of potential drugs.

Bipolar disorder is characterized by mood swings that affect a person’s quality of life. In this study, some drugs used in the treatment of this disorder are modeled using graphs, various versions of Zagreb indices are calculated, and equations are derived between the boiling points, enthalpies of vaporization, flash points, molar refractivity, and polarizability properties of these drugs and topological indices. The equations and indices with the best predictive ability are determined by examining the correlation between the properties of the drugs under investigation and the indices.

In this study, various Zagreb-type topological indices were applied to model the physicochemical properties of drugs used in the treatment of bipolar disorder. The results demonstrate that topological descriptors can effectively predict properties such as boiling point, enthalpy of vaporization, flash point, molar refractivity, and polarizability. Among the considered indices, the edge eccentric first Zagreb index ($EM_{1}^*$) was identified as the most effective predictor for boiling point, enthalpy of vaporization, and flash point. For molar refractivity and polarizability, the modified second Zagreb index ($MoM_{2}$) provided the best predictive performance.

A key contribution of this study is the demonstration that edge-based eccentric Zagreb indices can serve as powerful descriptors in QSPR modeling of pharmaceutical compounds. This highlights their potential application in computer-aided drug design. Furthermore, the results show that nonlinear regression models, particularly cubic models, generally provide better predictive accuracy. However, simpler models may still be preferred due to their interpretability.

The literature does not contain any information regarding studies on the topological indices of drugs used in the treatment of bipolar disorder. Therefore, the results of this study will guide the discovery of new drugs for the treatment of bipolar disorder and will also contribute to research in the field of mathematical chemistry.

## Data Availability

All data generated or analysed during this study are included in this published article. The data used and analysed during the current study available from the corresponding author on reasonable request.
